# The effects on weight loss and gene expression in adipose and hepatic tissues of very-low carbohydrate and low-fat isoenergetic diets in diet-induced obese mice

**DOI:** 10.1186/s12986-016-0139-1

**Published:** 2016-11-08

**Authors:** Tomomi Yamazaki, Sumire Okawa, Mayumi Takahashi

**Affiliations:** 1 Department of Nutritional Science, National Institute of Health and Nutrition, National Institutes of Biomedical Innovation, Health and Nutrition, 1-23-1 Toyama, Shinjuku-ku, Tokyo, 162-8636 Japan; 2 Department of Life Science, Osaka Women’s Junior College, 3-8-1 Kasugaoka, Fujiidera City, Osaka 583-8558 Japan

**Keywords:** Very-low carbohydrate diet, Low-fat diet, Diet-induced obese mice, Weight loss, Fatty liver, Browning of WAT, Ketone bodies, FGF21, GPR120

## Abstract

**Background:**

Obesity is caused by excessive fat or carbohydrate intake. The improvement of obesity is an important issue, especially in Western societies. Both low-carbohydrate diet (LCD) and low-fat diet (LFD) are used to achieve weight loss in humans. To clarify the mechanisms underlying LCD-induced weight loss, especially in early stage, we compared the gene expression in liver, white adipose tissue (WAT) and brown adipose tissue (BAT) of a very-low carbohydrate diet (VLCD)- and LFD-fed diet-induced obese (DIO) mice.

**Methods:**

DIO male ddY mice were divided into high-fat diet (HFD), and isoenergetic VLCD and LFD groups. Pair-feeding was performed in the VLCD and LFD groups. Three weeks later, the body, liver, WAT and BAT were weighed and the serum and hepatic lipids, the mRNA expression levels in each tissue, and energy metabolism were analyzed.

**Results:**

The caloric intake of the VLCD-fed mice was initially reduced but was subsequently restored. The total energy intake was similar in the VLCD- and LFD-fed mice. There was a similar decrease in the BW of the VLCD- and LFD-fed mice. The VLCD-fed mice had elevated levels of serum fibroblast growth factor 21 (FGF21) and ketone bodies, which are known to increase energy expenditure. The browning of WAT was observed to a greater extent in the VLCD-fed mice. Moreover, in the VLCD-fed mice, BAT activation was observed, the weight of the BAT was decreased, and the expression of G-protein-coupled receptor 120, type 2 iodothyronine deiodinase, and FGF21 in BAT was extremely increased. Although the energy expenditure of the VLCD- and LFD-fed mice did not differ, that of the VLCD-fed mice was sometimes higher during the dark cycle. Hepatic TG accumulation was reduced in LFD-fed mice due to their decreased fatty acid uptake but not in the VLCD-fed mice. The pro-inflammatory macrophage ratio was increased in the WAT of VLCD-fed mice.

**Conclusions:**

After 3 weeks, the isoenergetic VLCD- and LFD-fed DIO mice showed similar weight loss. The VLCD-fed mice increased serum concentration of FGF21 and ketone bodies, and marker mRNA levels of browning in WAT, activation in BAT and hepatic lipogenesis.

## Background

The prevalence of obesity in Western societies, which is associated with increased fat or carbohydrate consumption, has increased dramatically [1–3]. The numerous clinical consequences of obesity include nonalcoholic fatty liver disease (NAFLD) [4], type 2 diabetes [5–8], and coronary heart disease [9]. The increase in obesity underlies the urgent need to test the safety and efficacy of widely-used weight-loss diets.

The low-carbohydrate diet (LCD) has increasingly gained attention as an alternative to the conventional low-fat diet (LFD) achieving effective weight loss and lowering serum triglyceride (TG) and insulin concentrations [10, 11]. However, there are conflicting reports of the weight loss and the changes in health-related biomarkers that occur with these diets. Systematic reviews and meta-analyses of the weight-loss effects and blood lipid and glucose level changes that occur with these diets have been reported. They suggested that an LCD of up to 6 months in duration might be a feasible alternative to an LFD for achieving weight loss and reducing the risk of cardiovascular disease; however, these effects are controversial at 12 months [12–14]. A recent study showed no differences at two years when overweight and obese adults were randomly assigned to undergo LCD and isoenergetic weight loss diets [15].

The effects of a very-low carbohydrate diet (VLCD)-fed C57BL/6 mice (ketogenic diet) revealed that weight loss was caused by increased energy expenditure; however, the studies compared obese HFD-fed mice with post-weight-loss VLCD-fed mice [16, 17]. VLCD-fed C57BL/6 mice remain lean, euglycemic, and hypoinsulinemic for longer but accumulate hepatic lipids [16]. In contrast, LCD-fed ob/ob mice exhibit persistent weight gain in spite of normalized fasting glycemia and reduced insulin and lipid levels [18, 19]. The analysis from the liver obtained from the VLCD-fed diet-induced obese (DIO) mice revealed that mRNA levels of sterol regulatory element-binding protein (SREBP)-1c, a transcriptional factor by which *de novo* lipogenesis is stimulated, and the target genes, such as fatty acid synthase (FAS), stearoyl-CoA desaturase 1 (SCD1), were decreased compared with the high-fat diet (HFD)-fed DIO mice [16]. There is no information about the expression of peroxisome proliferator-activated receptor (PPAR)α, a transcription factor responsible for fatty acid oxidation. The mice fed an HFD markedly increase the fatty acid intake of the liver and cause NAFLD, due to the elevated expression of PPARγ2 and the target genes [20]. NAFLD can be considered as an early predictor of metabolic disorders and type 2 diabetes; particularly in the normal-weight Asians and Caucasians [21, 22].

VLCD-fed non-obese C57BL/6J mice were reported to increase mRNA level of fibroblast growth factor 21 (FGF21), one of the hepatokines, in the liver rather than normal chow-fed mice [17]. Moreover, VLCD-fed non-obese C57BL/6J mice increased the gene expression levels of inflammatory factors in the liver [23]. Otherwise, these mice were reported to show a decrease on the levels of mRNA relating to the inflammation in white adipose tissue (WAT) [23]. WAT is an important endocrine organ which secretes adipokines, such as adiponectin and leptin. The expression of adiponectin in WAT is activated during adipogenesis, but it is significantly reduced in WAT from obese mice and humans [24]. Leptin, the product of the ob gene, is induced in WAT under the HFD [25]. Obesity is associated with chronic, low-grade inflammation. In fact, the expression of monocyte chemoattractant protein (MCP)-1 is increased in WAT in DIO mice and MCP-1 protein is considerd as one of the key chemokines that regulate migration and infiltration of macrophages [26]. Recently inducible ‘brown-like’ adipocytes, also known as ‘beige’ cells, have been reported to develop in WAT in response to various activators. These cells express a broad gene program that is distinct from either white or classical brown adipose tissue (BAT) which dissipates energy to produce heat and plays an importnant role in the regulation of energy balance [27, 28]. Promoting the development of brown or beige adipose tissue may protect against obesity and related metabolic features.

There are a few reports about the gene expression in the tissue from DIO mice after VLCD feeding as follows: liver [16, 23] and WAT [23]. Furthermore, no reports documenting the differences in the levels of mRNA in the liver, WAT and BAT between VLCD- and LFD- fed DIO mice have been published.

Therefore, we aimed to investigate the gene expression in liver, WAT, and BAT of DIO mice after isoenergetic VLCD and LFD feeding to clarify the systemic effects caused by VLCD feeding, especially in early stage. Moreover, we investigated the effects of these two diets on serum chemicals and also compared energy expenditure in similarly-weighted VLCD- and LFD-fed mice.

## Methods

### Animals

Six-week-old male ddY (*n* = 24) mice were obtained from Japan SLC, Inc. (Hamamatsu, Japan) and fed a normal laboratory diet (CE2; This diet is a GLP-compliant, standard rodent diet consisting mainly of vegetable protein with a proper balance of animal protein, Clea, Tokyo, Japan) for 1 week to stabilize metabolic conditions. Mice were exposed to a 12-h light/12-h dark cycle, and the room was maintained at a constant temperature of 22 °C. They were individually housed and allowed free access to experimental diets and water. Mice were cared for in accordance with the NIH Guide for the Care and Use of Laboratory Animals. All animal procedures were reviewed and approved by the National Institute of Health and Nutrition (No. 1311).

### Diets

Seven-week-old ddY mice were fed the HFD consisting of 60 energy% (en%) fat for 8 weeks to generate DIO. Eight mice each were subsequently assigned to one of three groups (HFD, VLCD, and LFD, *n* = 8 in each group). The diet preparations were similar to those of our previous studies [29, 30]. Detailed compositions of the experimental diets are listed in Table 1. The VLCD and the LFD contained 0.4 and 70 en% carbohydrate, respectively. VLCD chow was frozen once and cut up before feeding. The HFD-fed mice had *ad libitum* access to food for three more weeks. A 3-week pair-feeding study was performed, in which LFD-fed mice were fed an equal amount of food to that which the VLCD-fed mice consumed on the previous day. It is because the VLCD- and LFD-fed mice decreased their food intake in an early stage but the VLCD-fed mice showed a lower food intake than the LFD-fed mice. The food was provided to mice every day. Food intake per day was estimated by subtracting the food weight of that day from the initial food weight of the previous day. With these data, average energy intakes during total experimental periods in each group of mice were calculated.

**Table 1:** The dietary composition of experimental diets

Component	HFD	VLCD	LFD
	g / 100 g		
Safflower oil	8.4	18.3	4.0
Butter	25.2	54.8	–
Casein	26.4	8.1	20.0
α-Cornstarch	26.3	0.7	66.2
Vitamin mixture (AIN-93)	1.4	1.9	1.0
Mineral mixture (AIN-93)	4.9	6.6	3.5
Cellulose powder	7.0	9.4	5.0
L-Cystine	0.42	0.56	0.30

*HFD* high-fat diet, *VLCD* very-low carbohydrate diet, *LFD* low-fat diet

### Quantitative RT-PCR

The mice were euthanized by cervical dislocation, and the liver, epididymal WAT (eWAT), mesenteric WAT, subcutaneous WAT (sWAT) and BAT were isolated for RNA preparation in the morning from 3-h fasted animals to avoid acute effects of food intake. Total RNA isolated from the tissues was reverse transcribed with ReverTra Ace (Toyobo Co., Ltd., Osaka, Japan) with random hexamers. The resulting cDNA was PCR amplified in the 96-well format with SYBR Green PCR Master Mix and a 7500 Real-Time PCR System (Applied Biosystems, Foster City, CA). Expression levels of test genes were normalized to those of an endogenous control, acidic ribosomal phosphoprotein P0 (36B4). The level of 36B4 was invariable among samples of all experiments. The relative expression levels were calculated according to the formula 2^-∆Ct^, where ∆Ct is the difference in threshold cycle (Ct) values between the target and 36B4 endogenous control. The primers used for quantitative RT-PCR are listed in the previous report [20, 27, 29, 31] and in Table 2.

**Table 2:** The primers used for quantitative PCR

Gene	Forward primer (5' to 3')	Reverse primer (5' to 3')
*36B4*	GGCCCTGCACTCTCGCTTTC	TGCCAGGACGCGCTTGT
*Adiponectin*	AAGAAGGACAAGGCCGTTCTCTT	GCTATGGGTAGTTGCAGTCAGTT
*Arg-1*	CTCCAAGCCAAAGTCCTTAGAG	AGGAGCTGTCATTAGGGACATC
*BDH*	AGTTTGGGGTCGAGGCTTTC	TGGTGGCCGCTATGAAGTTG
*CD36*	AATGGCACAGACGCAGCCT	GGTTGTCTGGATTCTGGA
*CD68antigen*	TGACCTGCTCTCTCTAAGGCTACA	TCACGGTTGCAAGAGAAACATG
*CD137*	CGTGCAGAACTCCTGTGATAAC	GTCCACCTATGCTGGAGAAGG
*D2*	GCACGTCTCCAATCCTGAAT	TGAACCAAAGTTGACCACCA
*Elovl6*	TTCCGAGTCTCCCGGAAGT	ACAGCCCATCAGCATCTGAGT
*F4/80*	TGACAACCAGACGGCTTGTG	GCAGGCGAGGAAAAGATAGTGT
*FAS*	GCTGCGGAAACTTCAGGAAAT	AGAGACGTGTCACTCCTGGACTT
*FGF21*	ATGGAATGGATGAGATCTAGAGTTGG	TCTTGGTCGTCATCTGTGTAGAGG
*GRP120*	GCATAGGAGAAATCTCATGG	GAGTTGGCAAACGTGAAGGC
*HADH*	ACTACATCAAAATGGGCTCTCAG	AGCAGAAATGGAATGCGGACC
*HMGCS2*	ATCAACTCCCTGTGCCTGAC	GCAATGTCACCACAGACCAC
*Leptin*	GACACCAAAACCCTCAT	CAGAGTCTGGTCCATCT
*MCAD*	GATCGCAATGGGTGCTTTTGATAGAA	AGCTGATTGGCAATGTCTCCAGCAAA
*MCP-1*	CTTCTGGGCCTGCTGTTCA	CCAGCCTACTCATTGGGATCA
*Mincle*	ACCAAATCGCCTGCATCC	CACTTGGGAGTTTTTGAAGCATC
*MR*	CCACAGCATTGAGGAGTTTG	ACAGCTCATCATTTGGCTCA
*NOS2*	CAGCTGGGCTGTACAAACCTT	CATTGGAAGTGAAGCGTTTCG
*PGC1α*	AAGTGTGGAACTCTCTGGAACTG	GGGTTATCTTGGTTGGCTTTATG
*PPARα*	CCTCAGGGTACCACTACGGAGT	GCCGAATAGTTCGCCGAA
*PPARγ1*	GAGTGTGACGACAAGATTTG	GGTGGGCCAGAATGGCATCT
*PPARγ2*	TCTGGGAGATTCTCCTGTTGA	GGTGGGCCAGAATGGCATCT
*SCD1*	CCCCTGCGGATCTTCCTTAT	AGGGTCGGCGTGTGTTTCT
*SREBP-1c*	GGAGCCATGGATTGCACATT	CCTGTCTCACCCCCAGCATA
*TBX1*	GGCAGGCAGACGAATGTTC	TTGTCATCTACGGGCACAAAG
*TMEM26*	ACCCTGTCATCCCACAGAG	TGTTTGGTGGAGTCCTAAGGTC
*UCP1*	GGCCCTTGTAAACAACAAAATAC	GGCAACAAGAGCTGACAGTAAAT
*UCP2*	ACCAAGGGCTCAGAGCATGCA	TGGCTTTCAGGAGAGTATCTTTG

*36B4* acidic ribosomal phosphoprotein P0, *Arg* arginase, *BDH* 3-hydroxybutyrate dehydrogenase, *CD36* fatty acid translocase, *D2* type 2 iodothyronine deiodinase, *Elovl6* elongation of very long-chain fatty acid 6, *FAS* fatty acid synthase, *FGF21* fibroblast growth factor 21, *GPR* G-protein-coupled receptor, *HADH* hydroxyacyl-coenzyme A dehydrogenase, *HMGCS2* 3-hydroxy-3-methylglutaryl-coenzyme A synthase 2, *MCAD* medium-chain acyl-CoA dehydrogenase, *MCP* monocyte chemoattractant protein, *Mincle* the macrophage inducible C-type lectin, *MR* mannose receptor, *NOS* nitric oxide synthase, *PGC* peroxisome proliferator-activated receptor γ coactivator, *PPAR* peroxisome proliferator-activated receptor, *SCD1* stearoyl-CoA desaturase 1, *SREBP* sterol regulatory element-binding protein, *TBX1* T-box 1 transcription factor C, *TMEM26* transmembrane protein 26, *UCP* uncoupling protein

### Serum chemistries

The serum was obtained from the blood after standing for 30 min on ice and centrifugation for 15 min at 3,000 rpm at 4 °C. The serum glucose, TG, total cholesterol (TC), insulin, leptin, and adiponectin levels were determined as described previously [29, 32]. Serum glucose was measured on the Ascensia autoanalyzer (Bayer Corp., Tokyo, Japan). Serum TG and TC were assayed enzymatically using colorimetric kits, TG E test and cholesterol E test (Wako Pure Chemicals, Osaka, Japan). Serum insulin, leptin, and adiponectin were determined by the mouse insulin ELISA kit (Morinaga, Kanagawa, Japan), the mouse leptin ELISA kit (Morinaga), and mouse/rat adiponectin ELISA kit (Otsuka pharmaceutical Co, Tokyo, Japan), respectively. FGF21 was determined using a mouse FGF-21 DuoSet ELISA kit (R&D Systems, Inc., Minneapolis, MN). Serum ketone body levels were determined using an Autokit Total Ketone Bodies assay (Wako Pure Chemical Industries, Ltd., Osaka, Japan).

### Liver and feces analyses

Lipids in the liver and feces were extracted quantitatively with an ice-cold mixture of chloroform and methanol (2:1, v/v) by the method of Folch et al. [33]. After the organic phase was dried, extracted TG and TC in the liver and feces were measured as described above [32]. Liver glycogen was extracted and digested to glucose to determine its quantity using anthrone method as described previously [34].

### Hepatic histology

Mouse livers were fixed in 4 % neutral-buffered formalin, embedded in paraffin, cut into sections, and stained with Oil red O as previously described [29]. For Oil red O staining, a stock solution of Oil Red O (0.5 g/100 mL) in isopropanol was prepared, stored, and protected from light.

### Measurement of oxygen consumption and carbon dioxide production

Open-circuit indirect calorimetry was performed with an O_2_/CO_2_ metabolism measuring system for small animals (MK-5000RQ; Muromachi Kikai, Tokyo, Japan). The system monitored oxygen consumption (VO_2_) and carbon dioxide production (VCO_2_) at 3-min intervals and calculated the respiratory quotient (RQ) ratio (VCO_2_/VO_2_). Spontaneous motor activity was measured using the Supermex infrared sensor (Muromachi Kikai). Measurements were performed for the dark (from 19:00 to 7:00) or light (from 7:00 to 16:30) period under *ad libitum* feeding conditions. The substrate utilization rate and energy production rate were calculated using the formula used by Ferrannini [35] where the rate of glucose oxidation (g/min) = 4.55VCO_2_ (L/min)-3.21VO_2_(L/min)-2.87 N(mg/min), the rate of lipid oxidation (g/min) = 1.67 (VO_2_-VCO_2_)-1.92N, and the rate of energy production (kcal/min) = 3.91VO_2_ + 1.10VCO_2_-3.34N, where N is the rate of urinary nitrogen excretion used to estimate protein oxidation. However, considering that only a small portion of resting and exercise energy expenditure arises from protein oxidation [36], the contributions of protein oxidation were neglected.

### Statistical analysis

A one-way analysis of variance was performed (StatView Version 5.0, Abacus Concepts, Inc., Berkeley, CA) for the inter-group comparisons. Statistically significant variables were compared by Fisher’s protected least significance test. Statistical significance was set at *P* < 0.05.

## Results

### Food intake, weight loss, and body composition in the VLCD and LFD groups

**Fig. 1:**
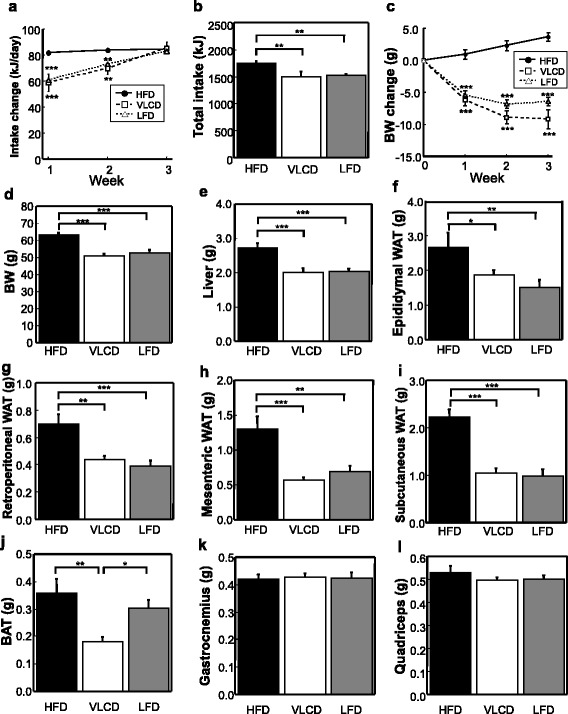
Daily and total energy intake, BW change, and body and tissue weights. (**a**) Daily energy intake. Food intake was measured daily during the study and weekly averages were calculated. (**b**) Total energy intake. (**c**) BW change over 3 weeks. The weights of the body (**d**), liver (**e**), epididymal (**f**), retroperitoneal (**g**), mesenteric (**h**), and subcutaneous WAT (**i**), BAT (**j**), gastrocnemius (**k**), and quadriceps (**l**). HFD, high-fat diet; VLCD, very-low carbohydrate diet; LFD, low-fat diet. Values are the mean ± SEM (*n* = 8). *P < 0.05, **P < 0.01, ***P < 0.001

The ddY mice feeding HFD for 8 weeks became obese (58.9 ± 0.6 g, *n* = 24). Thereafter, they were divided into 3 groups, HFD, VLCD and LFD group (*n* = 8 in each group). For the first week, the energy intake of the VLCD-fed mice was spontaneously reduced by 28 % (*P* < 0.001). However, their food intake gradually increased and was not reduced in the final week (Fig. 1a). The total energy intake was similar in the VLCD- and LFD-fed mice (Fig. 1b). The body weights (BWs) of the HFD-fed mice increased to 106 % at 3 weeks. There was a significant decrease in the BWs of the VLCD- and LFD-fed mice (14 and 11 %, respectively, *P* < 0.001 for both groups vs HFD); the difference in the rate of decrease was non-significant (Fig. 1c and d). The weights of the liver (HFD, 2.710 ± 0.153 g; VLCD, 1.989 ± 0.147 g; LFD, 2.040 ± 0.064 g; HFD vs VLCD, *P* < 0.001; HFD vs LFD, *P* < 0.001), eWAT (HFD, 2.665 ± 0.405 g; VLCD, 1.864 ± 0.125 g; LFD, 1.511 ± 0.222 g; HFD vs VLCD, *P* < 0.05; HFD vs LFD, *P* < 0.01), retroperitoneal WAT (HFD, 0.699 ± 0.072 g; VLCD, 0.439 ± 0.024 g; LFD, 0.388 ± 0.046 g; HFD vs VLCD, *P* < 0.01; HFD vs LFD, *P* < 0.001), mesenteric WAT (HFD, 1.302 ± 0.154 g; VLCD, 0.573 ± 0.035 g; LFD, 0.698 ± 0.080 g; HFD vs VLCD, *P* < 0.001; HFD vs LFD, *P* < 0.01), and sWAT (HFD, 2.231 ± 0.171 g; VLCD, 1.045 ± 0.094 g; LFD, 0.979 ± 0.153 g; HFD vs VLCD, *P* < 0.001; HFD vs LFD, *P* < 0.001) were significantly lower in the VLCD- and LFD-fed mice than in the HFD-fed mice (Fig. 1e-i). The BAT weight was significantly lower in the VLCD-fed mice than in the HFD- and LFD-fed mice (HFD, 0.359 ± 0.049 g; VLCD, 0.179 ± 0.019 g; LFD, 0.303 ± 0.029 g; HFD vs VLCD, *P* < 0.01; VLCD vs LFD, *P* < 0.05; Fig. 1j). The weights of the gastrocnemius and quadriceps were similar in all groups (Fig. 1k and l).

### Hepatic analyses

**Fig. 2:**
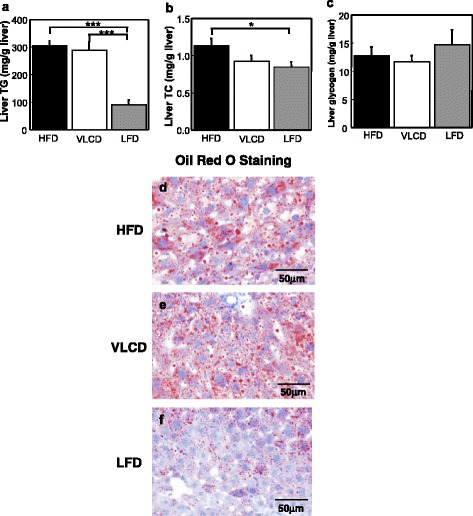
The hepatic analyses. (**a**) Liver TG, (**b**) liver TC, (**c**) liver glycogen content, and Oil red O staining of liver sections from mice fed (**d**) HFD, (**e**) VLCD, and (**f**) LFD. HFD, high-fat diet; VLCD, very-low carbohydrate diet; LFD, low-fat diet. Values are the mean ± SEM (*n* = 8). *P < 0.05, ***P < 0.001

Although there was a dramatic decrease in the BWs of the mice in the VLCD and LFD groups at 3 weeks (HFD, 63.088 ± 1.080 g; VLCD, 51.925 ± 1.107 g; LFD, 52.638 ± 1.686 g; HFD vs VLCD, *P* < 0.001; HFD vs LFD, *P* < 0.001; Fig. 1d), the effects of these diets on NAFLD were different. The liver TG content of the VLCD-fed mice was equal to that in the HFD-fed mice, indicating a same increase compared to the HFD-fed mice at 3 weeks (Fig. 2a). In contrast, hepatic TG and TC accumulation were markedly decreased in the LFD-fed mice compared to the HFD- fed mice (HFD vs LFD, *P* < 0.001; VLCD vs LFD, *P* < 0.001 for TG; HFD vs LFD, *P* < 0.05 for TC; Fig. 2a and b). The liver glycogen content of the VLCD-fed mice was almost the same as that of the HFD- and LFD-fed mice, indicating that glycogen synthesis was sufficient despite the lack of carbohydrates in the diet (Fig. 2c). Oil red O staining confirmed hepatic TG accumulation in both the HFD- and VLCD-fed mice but not in LFD-fed mice (Fig. 2d-f).

### Serum and feces analyses

**Fig. 3:**
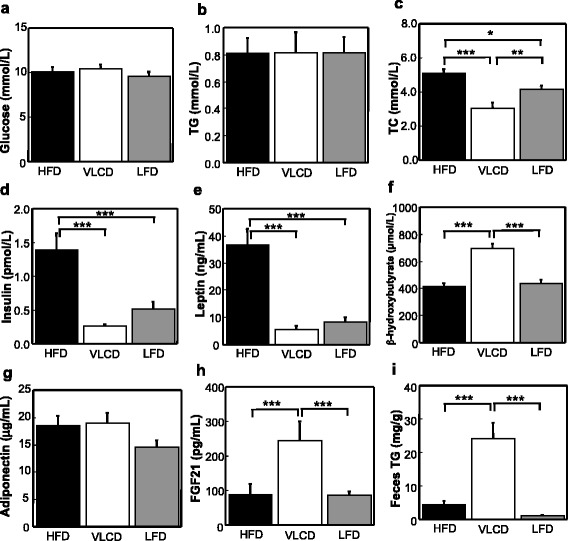
The serum and feces analyses. (**a**) serum glucose, (**b**) serum TG, (**c**) serum TC, (**d**) serum insulin, (**e**) serum leptin, (**f**) serum β-hydroxybutyrate, (**g**) serum adiponectin, (**h**) serum FGF21 concentration, (**i**) feces TG content. HFD, high-fat diet; VLCD, very-low carbohydrate diet; LFD, low-fat diet. Values are the mean ± SEM (*n* = 8). *P < 0.05, **P < 0.01, ***P < 0.001

The serum glucose and TG concentrations of the HFD-, VLCD- and LFD-fed mice were the same (Fig. 3a and b). The serum TC concentration was lowest in the VLCD-fed mice, while that in the LFD-fed mice was also lower than in the HFD-fed mice (HFD, 5.11 ± 0.25 mmol/L; VLCD, 3.05 ± 0.03 mmol/L; LFD, 4.15 ± 0.24 mmol/L; HFD vs VLCD, *P* < 0.001; HFD vs LFD, *P* < 0.05; VLCD vs LFD, *P* < 0.01; Fig. 3c). The serum insulin and leptin concentrations were significantly decreased in the VLCD- and LFD-fed mice (insulin; HFD, 1.39 ± 0.25 pmol/L; VLCD, 0.27 ± 0.03 pmol/L; LFD, 0.52 ± 0.10 pmol/L; HFD vs VLCD, *P* < 0.001; HFD vs LFD, *P* < 0.001; leptin; HFD, 36.84 ± 5.99 ng/mL; VLCD, 5.39 ± 1.60 ng/mL; LFD, 8.37 ± 1.73 ng/mL; HFD vs VLCD, *P* < 0.001; HFD vs LFD, *P* < 0.001; Fig. 3d and Fig. 3e). The serum β-hydroxybutyrate (major circulating ketone) level was significantly elevated in the VLCD-fed mice compared to the HFD- and LFD-fed mice (HFD, 414 ± 24 μmol/L; VLCD, 694 ± 34 μmol/L; LFD, 436 ± 32 μmol/L; HFD vs VLCD, *P* < 0.001; VLCD vs LFD, *P* < 0.001; Fig. 3f). There was no difference in the serum adiponectin concentration (Fig. 3g). The serum FGF21 concentration was significantly increased in the VLCD-fed mice (HFD, 90.1 ± 29.1 pg/mL; VLCD, 245.7 ± 54.5 pg/mL; LFD, 84.7 ± 12.2 pg/mL; HFD vs VLCD, *P* < 0.001; VLCD vs LFD, *P* < 0.001; Fig. 3h). The fecal TG content was significantly increased in the VLCD-fed mice (HFD, 4.41 ± 1.27 mg/g; VLCD, 24.11 ± 4.88 mg/g; LFD, 1.18 ± 0.21 mg/g; HFD vs VLCD, *P* < 0.001; VLCD vs LFD, *P* < 0.001; Fig. 3i). It is estimated that 0.3, 1.1, and 0.4 % of the ingested fat was excreted as feces in the HFD-, VLCD-, and LFD-fed mice, respectively.

### Hepatic mRNA expression in HFD-, VLCD- and LFD-fed mice

**Fig. 4:**
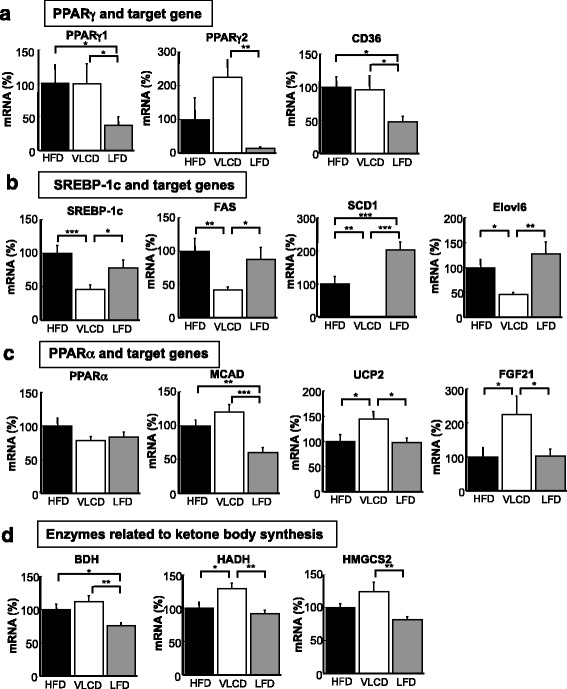
The hepatic gene expression. (**a**) PPARγ and CD36, (**b**) SREBP-1c and target genes, (**c**) PPARα and target genes, and (**d**) enzymes related to ketone body synthesis. The percentages of mRNA levels relative to those of HFD-fed mice are shown. HFD, high-fat diet; VLCD, very-low carbohydrate diet; LFD, low-fat diet. Values are the mean ± SEM (*n* = 8). *P < 0.05, **P < 0.01, ***P < 0.001

The decrease in the liver TG content of the LFD-fed mice, led us to investigate mRNA expression in the liver of HFD-, VLCD- and LFD-fed mice. We previously reported that an HFD increased PPARγ2 mRNA expression in mice [20]. There was a decrease in the mRNA levels of PPARγ1, γ2, and fatty acid translocase (CD36: a target gene of PPARγ) in the LFD-fed mice; however, the VLCD-fed mice showed the highest PPARγ2 level (PPARγ1; HFD, 100 ± 28; VLCD, 99 ± 30; LFD, 38 ± 12; HFD vs LFD, *P* < 0.05; VLCD vs LFD, *P* < 0.05; PPARγ2; HFD, 100 ± 65; VLCD, 224 ± 53; LFD, 14 ± 5; VLCD vs LFD, *P* < 0.01; CD36; HFD, 100 ± 16; VLCD, 96 ± 21; LFD, 48 ± 8; HFD vs LFD, *P* < 0.05; VLCD vs LFD, *P* < 0.05; Fig. 4a). On the other hand, there was a significant decrease in the expression of SREBP-1c in the VLCD-fed mice compared to the HFD- and LFD-fed mice (HFD, 100 ± 12; VLCD, 45 ± 7; LFD, 78 ± 11; HFD vs VLCD, *P* < 0.001; VLCD vs LFD, *P* < 0.05; Fig. 4b). SREBP-1c activation increases the expression of lipogenic genes, such as FAS, SCD1, and elongation of very long-chain fatty acid 6 (Elovl6) [20]. The levels of these mRNAs were also decreased in VLCD-fed mice (FAS; HFD, 100 ± 19; VLCD, 42 ± 4; LFD, 88 ± 17; HFD vs VLCD, *P* < 0.01; VLCD vs LFD, *P* < 0.05; SCD1; HFD, 100.0 ± 21.9; VLCD, 0.4 ± 0.1; LFD, 202.4 ± 24.4; HFD vs VLCD, *P* < 0.01; HFD vs LFD, *P* < 0.001; VLCD vs LFD, *P* < 0.001; Elovl6; HFD, 100 ± 16; VLCD, 46 ± 3; LFD, 128 ± 23; HFD vs VLCD, *P* < 0.05; VLCD vs LFD, *P* < 0.01; Fig. 4b). There were no differences in the expression of PPARα, of the dietary groups (Fig. 4c). However, the mRNA level of the medium-chain acyl-CoA dehydrogenase (MCAD) was significantly lower in the LFD-fed mice than in the other groups (HFD, 100 ± 8; VLCD, 120 ± 11; LFD, 60 ± 8; HFD vs LFD, *P* < 0.01; VLCD vs LFD, *P* < 0.001; Fig. 4c). The uncoupling protein (UCP)2 level was significantly increased in the VLCD-fed mice (HFD, 100 ± 13; VLCD, 145 ± 14; LFD, 97 ± 11; HFD vs VLCD, *P* < 0.05; VLCD vs LFD, *P* < 0.05; Fig. 4c). The FGF21 mRNA level was significantly increased in the VLCD-fed mice (HFD, 100 ± 27; VLCD, 225 ± 54; LFD, 101 ± 21; HFD vs VLCD, *P* < 0.05; VLCD vs LFD, *P* < 0.05; Fig. 4c), in accordance with the increase in the serum FGF21 concentration. As expected from the increase of serum ketone bodies in the VLCD-fed mice, the mRNA levels of 3-hydroxybutyrate dehydrogenase (BDH), hydroxyacyl-coenzyme A dehydrogenase (HADH) and 3-hydroxy-3-methylglutaryl-coenzyme A synthase 2 (HMGCS2), which are responsible for ketone metabolism, were increased in these mice (BDH; HFD, 100 ± 8; VLCD, 112 ± 9; LFD, 76 ± 4; HFD vs LFD, *P* < 0.05; VLCD vs LFD, *P* < 0.01; HADH; HFD 100 ± 10; VLCD, 129 ± 9; LFD, 92 ± 5; HFD vs VLCD, *P* < 0.05; VLCD vs LFD, *P* < 0.01; HMGCS2; HFD, 100 ± 6; VLCD, 124 ± 14; LFD, 81 ± 5; VLCD vs LFD, *P* < 0.01; Fig. 4d).

### Gene expression in the WAT of HFD-, VLCD- and LFD-fed mice

**Fig. 5:**
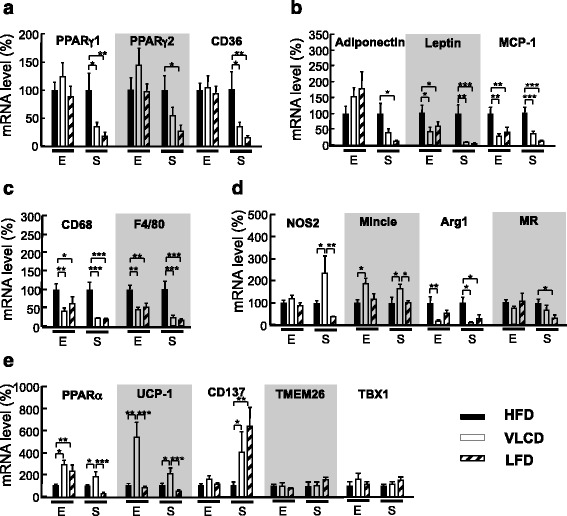
Gene expression in the epididymal and subcutaneous WAT. (**a**) PPARγ and CD36, (**b**) adipokines, (**c**) macrophage surface markers, (**d**) M1 and M2 macrophage markers, and (**e**) browning markers. The percentages of mRNA levels relative to those of HFD-fed mice are shown. E, Epididymal WAT; S, Subcutaneous WAT. HFD, high-fat diet; VLCD, very-low carbohydrate diet; LFD, low-fat diet. Values are the mean ± SEM (*n* = 8). *P < 0.05, **P < 0.01, ***P < 0.001

The body and WAT weights were significantly decreased in the VLCD- and LFD-fed mice. We therefore examined mRNA expression in the eWAT and sWAT. As shown in Fig. 5a, there were no significant differences in the PPARγ1, γ2 and CD36 levels in the eWAT of the three groups. However, these mRNAs were significantly decreased in the sWAT of LFD-fed mice, PPARγ1 and CD36 mRNAs were significantly decreased in the VLCD-fed mice. Adipokines, such as adiponectin and leptin, are secreted from WAT. There were no significant differences among the groups in the adiponectin mRNA levels in the eWAT (Fig. 5b). Leptin expression in the eWAT and sWAT was significantly lower in the VLCD- and LFD-fed mice than in the HFD-fed mice in accordance with the decrease in serum leptin concentration (eWAT; HFD, 100 ± 24; VLCD, 41 ± 13; LFD, 60 ± 15; HFD vs VLCD, *P* < 0.05; HFD vs LFD, *P* < 0.05; sWAT; HFD, 100 ± 28; VLCD, 10 ± 3; LFD, 6 ± 2; HFD vs VLCD, *P* < 0.01; HFD vs LFD, *P* < 0.001; Fig. 5b). The mRNA levels of MCP-1, one of the key chemokines that regulate migration and infiltration of macrophages, was significantly decreased in all WAT types in the VLCD- and LFD-fed mice (eWAT; HFD, 100 ± 21; VLCD, 27 ± 5; LFD, 37 ± 14; HFD vs VLCD, *P* < 0.01; HFD vs LFD, *P* < 0.01; sWAT; HFD, 100 ± 20; VLCD, 30 ± 8; LFD, 12 ± 2; HFD vs VLCD, *P* < 0.001; HFD vs LFD, *P* < 0.001; Fig. 5b). As expected from the decreased MCP-1 mRNA levels, the CD68 and F4/80 (macrophage markers) mRNA levels were decreased in the eWAT and sWAT of the VLCD- and LFD-fed mice (CD68 in eWAT; HFD, 100 ± 18; VLCD, 42 ± 8; LFD, 64 ± 20; HFD vs VLCD, *P* < 0.01; HFD vs LFD, *P* < 0.05; CD68 in sWAT; HFD, 100 ± 22; VLCD, 22 ± 4; LFD, 19 ± 3; HFD vs VLCD, *P* < 0.001; HFD vs LFD, *P* < 0.001; F4/80 in eWAT; HFD, 100 ± 12; VLCD, 46 ± 6; LFD, 53 ± 12; HFD vs VLCD, *P* < 0.01; HFD vs LFD, *P* < 0.01; F4/80 in sWAT; HFD, 100 ± 22; VLCD, 23 ± 7; LFD, 16 ± 5; HFD vs VLCD, *P* < 0.001; HFD vs LFD, *P* < 0.001; Fig. 5c). We next investigated the expression of pro-inflammatory M1 macrophage markers (nitric oxide synthase [NOS]2 and Mincle) and anti-inflammatory M2 macrophage markers (arginase [Arg]1 and mannose receptor [MR]) . NOS2 was increased in sWAT in the VLCD-fed mice (HFD, 100 ± 15; VLCD, 239 ± 78; LFD, 39 ± 4; HFD vs VLCD, *P* < 0.05; VLCD vs LFD, *P* < 0.01; Fig. 5d). The mRNA level of Mincle, the macrophage inducible C-type lectin, was increased in eWAT and sWAT in the VLCD-fed mice (eWAT; HFD, 100 ± 14; VLCD, 186 ± 27; LFD, 116 ± 26; HFD vs VLCD, *P* < 0.05; sWAT; HFD, 100 ± 27; VLCD, 141 ± 20; LFD, 101 ± 10; HFD vs VLCD, *P* < 0.05; VLCD vs LFD, *P* < 0.05; Fig. 5d). The expressions of Arg1 and MR were decreased in the sWAT of the LFD-fed mice (Arg1 in sWAT; HFD, 100 ± 28; VLCD, 12 ± 4; LFD, 30 ± 18; HFD vs VLCD, *P* < 0.05; HFD vs LFD, *P* < 0.05; MR in sWAT; HFD, 100 ± 21; VLCD, 68 ± 22; LFD, 33 ± 14; HFD vs LFD, *P* < 0.05; Fig. 5d). As for VLCD-fed mice, only the Arg1 mRNA levels were decreased in eWAT and sWAT (Arg1 in eWAT; HFD, 100 ± 31; VLCD, 20 ± 4; LFD, 56 ± 14; HFD vs VLCD, *P* < 0.01; Fig. 5d).

Beige/browning cells express UCP1 and a broad gene program that is distinct from either white or classical brown adipocytes [27]. Hence, we investigated the expression of UCP1 and other beige/browning markers. Increased UCP1 and PPARα mRNA levels were observed in the eWAT of VLCD-fed mice, while only PPARα was increased in LFD-fed mice (PPARα in eWAT; HFD, 100 ± 17; VLCD, 291 ± 45; LFD, 235 ± 58; HFD vs VLCD, *P* < 0.05; HFD vs LFD, *P* < 0.01; UCP1 in eWAT; HFD, 100 ± 28; VLCD, 547 ± 134; LFD, 88 ± 17; HFD vs VLCD, *P* < 0.01; VLCD vs LFD, *P* < 0.001; Fig. 5e). Increased PPARα and UCP1 mRNA levels were observed in the sWAT of VLCD-fed mice (PPARα in sWAT; HFD, 100 ± 19; VLCD, 194 ± 44; LFD, 18 ± 3; HFD vs VLCD, *P* < 0.05; VLCD vs LFD, *P* < 0.001; UCP1 in sWAT; HFD, 100 ± 28; VLCD, 212 ± 54; LFD, 25 ± 2; HFD vs VLCD, *P* < 0.05; VLCD vs LFD, *P* < 0.001; Fig. 5e). The increased expression of CD137, another beige/browning marker, was observed in the sWAT of VLCD- and LFD-fed mice (HFD, 100 ± 43; VLCD, 409 ± 187; LFD, 647 ± 171; HFD vs VLCD, *P* < 0.05; HFD vs LFD, *P* < 0.01; Fig. 5e). With regard to the other beige/browning markers (TMEM26 and TBX1), no significant difference was observed.

### Gene expression in the BAT in HFD-, VLCD- and LFD-fed mice

**Fig. 6:**
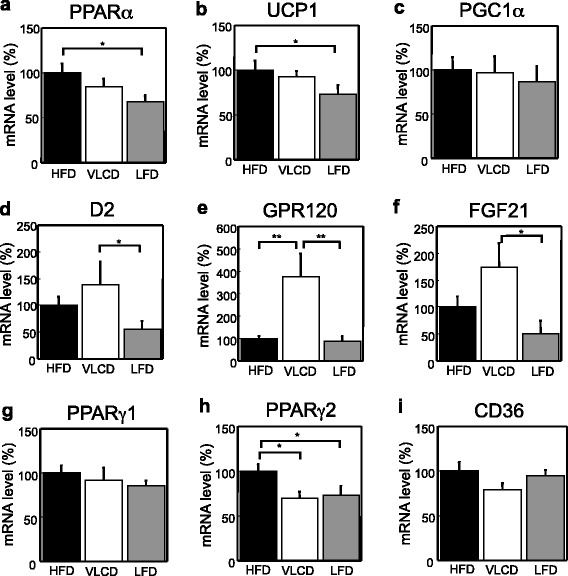
Gene expression in BAT. (**a**) PPARα, (**b**) UCP1, (**c**) PGC1α, (**d**) D2, (**e**) GPR120, (**f**) FGF21, (**g**) PPARγ1, (**h**) PPARγ2, and (**i**) CD36. The percentages of mRNA levels relative to those of HFD-fed mice are shown. HFD, high-fat diet; VLCD, very-low carbohydrate diet; LFD, low-fat diet. Values are the mean ± SEM (*n* = 8). *P < 0.05, **P < 0.01

In BAT, UCP1 is responsible for nonshivering thermogenesis [37]. The PPARα and UCP1 levels decreased in the LFD-fed mice (PPARα; HFD, 100 ± 11; VLCD, 85 ± 9; LFD, 68 ± 6; HFD vs LFD, *P* < 0.05; UCP1; 100 ± 10; VLCD, 93 ± 6; LFD, 73 ± 10; HFD vs VLCD, *P* < 0.05; Fig. 6a and b). The expression of the other UCP1 regulating factor, peroxisome proliferator-activated receptor γ coactivator (PGC)1α did not differ in the three groups (Fig. 6c). The mRNA level of type 2 iodothyronine deiodinase (D2), which converts T4 to T3, was increased in the VLCD-fed mice (HFD, 100 ± 17; VLCD, 140 ± 42; LFD, 56 ± 14; VLCD vs LFD, *P* < 0.05; Fig. 6d). Surprisingly, there was an extraordinary increase in the expression of G-protein-coupled receptor (GPR)120, which is activated by free fatty acid (FFA), in the VLCD-fed mice (HFD, 100 ± 13; VLCD, 375 ± 102; LFD, 88 ± 25; HFD vs VLCD, *P* < 0.01; VLCD vs LFD, *P* < 0.01; Fig. 6e). FGF21 mRNA was increased in the BAT similarly to the liver of VLCD-fed mice (HFD, 100 ± 20; VLCD, 174 ± 45; LFD, 50 ± 24; VLCD vs LFD, *P* < 0.05; Fig. 6f). There were no significant differences in PPARγ1 expression (Fig. 6g). PPARγ2 mRNA was significantly decreased in the VLCD- and LFD-fed mice (HFD, 100 ± 8; VLCD, 70 ± 7; LFD, 73 ± 11; HFD vs VLCD, *P* < 0.05; HFD vs LFD, *P* < 0.05; Fig. 6h). There were no significant differences in CD36 expression among the three groups (Fig. 6i).

### Substrate utilization in VLCD- and LFD-fed mice

**Fig. 7:**
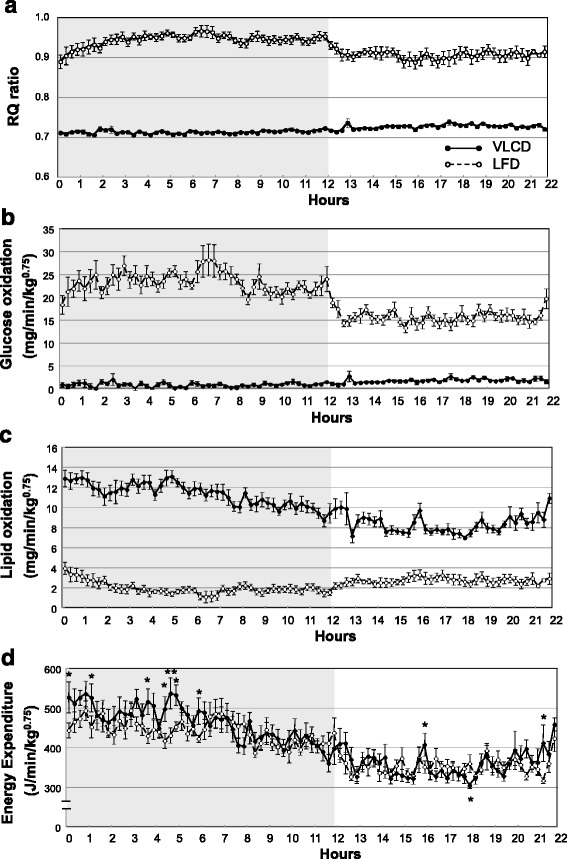
The respiratory quotient (RQ), glucose and lipid oxidation rates, and energy expenditure rates. (**a**) RQ ratio, (**b**) glucose oxidation rates, (**c**) lipid oxidation rates, and (**d**) energy expenditure rates in VLCD- and LFD-fed mice. Dark and light cycles are indicated by the gray and white backgrounds, respectively. Values are the mean ± SEM (*n* = 8). Statistical analysis was performed only for data from energy expenditure rates. *P < 0.05, **P < 0.01

Oxygen consumption was measured after 3 weeks in the VLCD- and LFD-fed mice. The oxygen consumption of VLCD-fed mice during the dark cycle (feeding period) was greater than that of LFD-fed mice (*P* = 0.030), but not during the light cycle (sleeping period) (Table 3). Carbon dioxide production and the RQ decreased in the VLCD-fed group during both the dark and light cycles (Fig. 7a and Table 3). The activity levels were higher in the LFD-fed mice than in VLCD-fed mice, but only in the dark cycle (Table 3). Throughout the day, glucose oxidation was higher in the LFD-fed mice than in the VLCD-fed mice (*P* < 0.001 at all observed time, Fig. 7b and Table 3). In contrast, lipid oxidation was higher in the VLCD-fed mice than in the LFD-fed mice (*P* < 0.001 at all observed time, Fig. 7c and Table 3). The energy production of the VLCD- and LFD-fed mice did not differ throughout the day; however, the VLCD-fed mice showed higher energy expenditure, especially early in the dark cycle (Fig. 7d and Table 3).

**Table 3:** The oxygen consumption, carbon dioxide production, RQ ratio and spontaneous motor activity

	VLCD	LFD	*P*
n	8	8	
Body weight (g)	50.0 ± 0.6	51.6 ± 1.3	0.180
Dark cycle			
VO_2_ (ml/min/kg^0.75^)	23.5 ± 0.9	21.3 ± 0.3	0.030
VCO_2_ (ml/min/kg^0.75^)	16.8 ± 0.6	20.1 ± 0.4	0.001
RQ	0.712 ± 0.001	0.944 ± 0.010	<0.001
Activity (count/min)	169 ± 10	234 ± 16	0.004
Glucose oxidation (mg/min/kg^0.75^)	0.78 ± 0.11	23.3 ± 1.1	<0.001
Lipid oxidation (mg/min/kg^0.75^)	11.3 ± 0.4	1.9 ± 0.3	<0.001
Energy production (J/min/kg^0.75^)	462 ± 17	441 ± 7	0.278
Light cycle			
VO_2_ (ml/min/kg^0.75^)	18.2 ± 0.8	17.2 ± 0.2	0.229
VCO_2_ (ml/min/kg^0.75^)	13.2 ± 0.5	15.5 ± 0.1	0.001
RQ	0.726 ± 0.002	0.905 ± 0.011	<0.001
Activity (count/min)	64.8 ± 6.9	70.4 ± 5.7	0.540
Glucose oxidation (mg/min/kg^0.75^)	1.6 ± 0.2	15.6 ± 0.7	<0.001
Lipid oxidation (mg/min/kg^0.75^)	8.33 ± 0.37	2.72 ± 0.33	<0.001
Energy production (J/min/kg^0.75^)	358 ± 15	352 ± 4	0.738

*LFD* low-fat diet, *RQ* respiratory quotient, *VLCD* very-low carbohydrate diet, *VO*_*2*_ oxygen consumption; VCO_2_, carbon dioxide production

## Discussion

We herein show that the BW of DIO mice decreased as long as their caloric intake was reduced in an early stage of amelioration of obesity, regardless of macronutrient composition. Moreover, the data in this study suggest that differences in the substrate utilization of the VLCD- and LFD-fed mice did not affect energy production. However, the gene expression levels in adipose and hepatic tissues and serum chemicals were different dependently on the macronutrient composition. Approximately 40 years ago it was reported that two types of diets, that were identical in caloric content (70 % carbohydrate, 20 % protein, and 10 % fat vs. 70 % fat, 20 % protein, 10 % carbohydrate) achieved similar BW loss in obese men [38]. Since then, the effects of altering the fat and carbohydrate content of hypocaloric diets have been examined and thereby reduced-calorie diets have been shown to result in clinically meaningful weight loss regardless of the macronutrients that are emphasized [39–41]. However, LCDs have increasingly gained attention as an effective means of achieving weight loss in humans [10, 11].

In humans, an LCD usually results in a decreased caloric intake [39, 42]. Ketone bodies (mainly β-hydroxybutyrate), which are known to be increased by an LCD, can act both orexigenically and anorexigenically [43]. In normo- or hypoglycemia, β-hydroxybutyrate exerts an anorexigenic role, thereby inducing a reduction in food intake [44, 45]. We demonstrated here that the serum β-hydroxybutyrate concentration was elevated in VLCD-fed mice, suggesting that the increased ketone bodies resulted in reduced appetite and subsequent BW loss.

In the present study, NAFLD, which was caused by the initial HFD, only improved in the LFD-fed mice - despite the BW decrease in the VLCD-fed mice. NAFLD is caused by an imbalance between TG synthesis and removal in the liver [46]. This is affected by multiple pathways, including the rate of FFA delivery to the liver, hepatic *de novo* lipogenesis, FFA oxidation, and TG secretion from the liver [46]. We demonstrated here that the hepatic expression levels of PPARγ2 and the target genes were higher in VLCD-fed mice than in LFD-fed mice (Fig. 4a). In contrast, the levels of SREBP-1c and the target genes responsible for *de novo* lipogenesis were higher in the liver of LFD-fed mice (Fig. 4b). SREBP-1c is required in the activation of hepatic *de novo* lipogenesis by glucose [47]. The data presented here indicate that *de novo* lipogenesis was not activated in the VLCD-fed mice because they could not utilize carbohydrates. Moreover, serum insulin was decreased in the VLCD- and LFD-fed mice, suggesting that insulin-stimulated *de novo* lipogenesis, mediated by SREBP-1c activation, did not act in the liver of these mice. Although the PPARα level was unchanged in the VLCD-fed mice, MCAD and UCP2 expression was increased in the liver, implicating the activation of PPARα; and indicating that fatty acid oxidation is activated due to the substantial increase in fatty acid uptake (Fig. 4c). Taken together, these results indicate that there was no decrease in the TG level in the liver of VLCD-fed mice due to the high level of fatty acid uptake in the liver and sustained high expression of PPARγ2 and the target genes, despite decreases in BW and serum insulin. It is now understood that NAFLD is a precursor of nonalcoholic steatohepatitis, which progresses to cirrhosis in up to 25 % of patients [48]. Moreover, NAFLD can be considered as an early predictor of metabolic disorders and type 2 diabetes [21, 22]. The fact that the hepatic TG level of the VLCD-fed mice did not decrease should be seriously considered when seeking to avoid metabolic syndrome and type 2 diabetes.

WAT is an important endocrine organ which secretes a large number of adipokines, such as leptin, which are involved in a variety of physiological and pathological processes [24, 25]. HFD-fed mice enlarges adipocytes, but a decrease in WAT mass results in a decrease in the size of these cells, which contributes to the improvement in insulin sensitivity and adipokine secretion [49]. We presented here that isoenergetic VLCD and LFD (regardless of their macronutrient composition) result in meaningful reductions in BW and WAT weight in DIO mice. Leptin is secreted by sWAT rather than visceral WAT [50]. This is in agreement with the results of our study as leptin mRNA was drastically decreased in the sWAT and serum of VLCD- and LFD-fed mice. The plasma concentration of adiponectin is inversely related to BW, and visceral WAT is a more active producer of adiponectin than sWAT [51]. However, the serum concentration of adiponectin in this study did not increase in the VLCD- and LFD-fed mice, in spite of their weight loss. This is attributed to a subtle increase in the expression of adiponectin in eWAT. MCP-1 increases in the mature adipocyte fraction of obese mice and promotes monocyte infiltration into the WAT; these monocytes then differentiate into adipose tissue macrophages (ATMs), which secrete additional chemokines and cytokines and exacerbate the pro-inflammatory environment [52]. There is considerable evidence that ATMs establish a vicious cycle that aggravates inflammatory changes in WAT, preceding or associating with ectopic lipid accumulation in obese individuals [53]. There are two polarized states of ATM: M1 (proinflammatory macrophages); and M2 (anti-inflammatory macrophages) [54]. M1 macrophages enhance the production of Mincle and proinflammatory cytokines [31], and generate NO via iNOS (*Nos2*) activation [55]. M2 macrophages increase the production of Arg1 [56], which blocks iNOS activity, and MR leading to participate in the blockade of inflammatory responses and the promotion of tissue repair [57]. In accordance with the decrease in their fat mass, the macrophage cell surface markers (CD68 and F4/80) were markedly decreased in the WAT of VLCD- and LFD-fed mice (Fig. 5c). However, the M1/M2 ratio increased in the VLCD-fed mice. During the course of obesity, ATMs exhibit a phenotypic switch from M2 to M1 polarization [58]. Thus, while the condition of VLCD-fed mice may be good at the time of evaluation, the increased M1 macrophages in the WAT might induce a poor metabolic state in the future.

Inducible beige/brown-like adipocytes have recently been reported to develop in WAT in response to various activators and act as heat-producing adipocytes [27]. Since then, several beige cells marker genes have been proposed. Beige adipocytes have a gene expression pattern that is distinct from WAT and BAT [59]. Beige adipocytes, which are found in small amounts, most frequently in the inguinal fat in rodents, express mainly UCP-1, CD137, TBX1, and TMEM26 [27, 60, 61]. PPARα and UCP1 were increased in the eWAT and sWAT of the VLCD-fed mice in our study. Although the validity of the markers is controversial, CD137 is reportedly selective for beige adipocytes [59]. CD137 expression was extremely increased in the sWAT of both VLCD- and LFD-fed mice. FGF21, a pleiotropic protein involved in glucose and lipid metabolism, is mainly secreted by the liver as a nutritionally-regulated hormone partly induced in a PPARα-dependent manner [62]. FGF21 was reported to promote WAT browning [63]. Thus, increased FGF21 in the serum of VLCD-fed mice seems to induce WAT browning. The mechanism underlying the increase in CD137 expression in the LFD-fed mice is currently unknown, but it was reported that the attenuation of M1 recruitment to the WAT is beneficial to WAT browning and the improvement of the metabolic phenotype [64]. This is the first report to show WAT browning in DIO mice that lost weight due to a hypocaloric diet.

Surprisingly, we observed a marked decrease in the BAT weight of VLCD-fed mice. In a previous study, the BAT of mice that were fed ketone ester was shown to weigh significantly less (probably due to a decrease in BAT fat stores) [65]. Thus, it seems that the increase in serum ketone bodies in the VLCD-fed mice caused a decrease in BAT weight. BAT was once thought to only have a functional role in rodents and human infants; however, the adult human BAT has recently been reported to consume more glucose per gram in response to mild cold exposure than any other tissue [66], which indicates that BAT is responsible for non-shivering thermogenesis. FGF21 has been reported to promote thermogenic activity in BAT [67]. Indeed, FGF21 was reported to reduce BW due to marked increases in total energy expenditure [68]. Moreover, thermogenic activation has been reported to induce a significant increase in the FGF21 mRNA levels of BAT [69]. In the present study, FGF21 mRNA was increased in the BAT of VLCD-fed mice (Fig. 6f). Thus, the increase in concentration of serum FGF21 and mRNA expression of FGF21 in BAT of VLCD-fed mice may demonstrate the thermogenic activation in part in our study. D2 is also induced for adaptive thermogenesis in the BAT [70]. In the present study, the BAT D2 mRNA level was highest in the VLCD-fed mice, indicating the activation of the BAT (Fig. 6d). Moreover, the BAT of ketone ester-fed mice was activated and there was a 14 % increase in their total energy expenditure [66]. These results indicate that the BAT of VLCD-fed mice was activated through an increase in serum ketone bodies and FGF21; however, energy expenditure did not differ from LFD-fed mice because of the subtle increase to clarify the differences in this study.

We described for the first time that GPR120 mRNA was extremely increased in the BAT of VLCD-fed mice. GPR120 is a member of the G protein-coupled receptors, which are expressed in the pancreas, intestine, adipocytes, and macrophages and are activated by FFA, especially medium to long-chain FFAs [71]. GPR120, which promotes glucagon-like peptide-1 secretion in the intestine [72], acts as a lipid sensor in adipose tissue to sense dietary fat and control energy balance [73]. Since BAT takes up lipolysis-mediated TG-derived FFA [74], GPR120 is likely responsible for some metabolic state changes in BAT, although the physiological function of GPR120 in BAT is unknown. Further studies on function of GPR120 in BAT are needed.

LCDs are used to treat neurological diseases such as epilepsy. Unrestricted LCDs have adverse effects on health-related biomarkers [75]. It was also suggested that longer-term studies are required to determine the long-term safety and efficacy of LCDs [41]. Taken together, LCD might be effective to lose weight for obese subjects for short-term because LCD decreases serum concentrations of insulin and leptin. However, these effects seem to be caused in part by the decreased appetite due to the increased serum ketone bodies. Moreover, LCD is not effective for amelioration of NAFLD. Therefore, employing LCD for weight loss for a long time should be carefully considered.

This study demonstrates that both a VLCD and an LFD for three weeks achieved BW loss and the improvement of metabolic parameters in DIO mice but that NAFLD was only improved by an LFD. Although the VLCD caused WAT browning, BAT activation, and increase in serum ketone bodies and FGF21, which might have in part, led to increased energy expenditure, the VLCD-fed mice seemed to use a “hidden power” to burn excess fat. The temporary reduction in the food intake of the VLCD-fed mice, the increased liver TG content and M1/M2 ratio in WAT should be carefully considered when VLCDs are employed on a long-term basis.

## Conclusion

In this study, we show here that the isoenergetic VLCD- and LFD-fed DIO mice showed similar weight loss in an early stage of amelioration of obesity, regardless of macronutrient composition. We also compared and show the differences in the levels of serum chemicals and mRNA in liver, WAT and BAT of the VLCD- and LCD-fed mice. The VLCD-fed mice increased serum ketone bodies and FGF21, and mRNA levels of browning makers in WAT and GPR120, D2, and FGF21 in BAT and these increases were considered, at least in part, to lead to their weight loss. However, NAFLD was only improved by LFD. Moreover, the VLCD-fed mice increased the M1/M2 ratio in WAT. Taken together, our study suggests that employing VLCDs for weight loss for a long time should be carefully considered.

## Abbreviations

36B4: Acidic ribosomal phosphoprotein P0
Arg: Arginase
ATM: Adipose tissue macrophage
BAT: Brown adipose tissue
BDH: 3-hydroxybutyrate dehydrogenase
BW: Body weight
CD36: Fatty acid translocase
Ct: Threshold cycle
D2: Type 2 iodothyronine deiodinase
DIO: Diet-induced obese
Elovl6: Elongation of very long-chain fatty acid 6
en%: Energy%
eWAT: Epididymal WAT
FAS: Fatty acid synthase
FFA: Free fatty acid
FGF21: Fibroblast growth factor 21
GPR: G-protein-coupled receptor
HADH: Hydroxyacyl-coenzyme A dehydrogenase
HFD: High-fat diet
HMGCS2: 3-hydroxy-3-methylglutaryl-coenzyme A synthase 2
LCD: Low-carbohydrate diet
LFD: Low-fat diet
MCAD: Medium-chain acyl-CoA dehydrogenase
MCP: Monocyte chemoattractant protein
Mincle: The macrophage inducible C-type lectin
MR: Mannose receptor
NAFLD: Nonalcoholic fatty liver disease
NOS: Nitric oxide synthase
PGC: Peroxisome proliferator-activated receptor γ coactivator
PPAR: Peroxisome proliferator-activated receptor
RQ: Respiratory quotient
SCD1: Stearoyl-CoA desaturase 1
SREBP: Sterol regulatory element-binding protein
sWAT: Subcutaneous WAT
TBX1: T-box 1 transcription factor C
TC: Total cholesterol
TG: Triglyceride
TMEM26: Transmembrane protein 26
UCP: Uncoupling protein
VCO_2_: Carbon dioxide production
VLCD: Very-low carbohydrate diet
VO_2_: Oxygen consumption
WAT: White adipose tissue.
